# Identifying Medication Management Smartphone App Features Suitable for Young Adults With Developmental Disabilities: Delphi Consensus Study

**DOI:** 10.2196/mhealth.9527

**Published:** 2018-05-23

**Authors:** Teresa M Salgado, Alexa Fedrigon, Donna Riccio Omichinski, Michelle A Meade, Karen B Farris

**Affiliations:** ^1^ Department of Pharmacotherapy & Outcomes Science School of Pharmacy Virginia Commonwealth University Richmond, VA United States; ^2^ College of Pharmacy University of Michigan Ann Arbor, MI United States; ^3^ Department of Physical Medicine & Rehabilitation University of Michigan Rehabilitation Engineering Research Center University of Michigan Ann Arbor, MI United States; ^4^ Department of Clinical Pharmacy College of Pharmacy University of Michigan Ann Arbor, MI United States

**Keywords:** developmental disabilities, intellectual disability, mobile applications, self-management, telemedicine, young adult

## Abstract

**Background:**

Smartphone apps can be a tool to facilitate independent medication management among persons with developmental disabilities. At present, multiple medication management apps exist in the market, but only 1 has been specifically designed for persons with developmental disabilities. Before initiating further app development targeting this population, input from stakeholders including persons with developmental disabilities, caregivers, and professionals regarding the most preferred features should be obtained.

**Objective:**

The aim of this study was to identify medication management app features that are suitable to promote independence in the medication management process by young adults with developmental disabilities using a Delphi consensus method.

**Methods:**

A compilation of medication management app features was performed by searching the iTunes App Store, United States, in February 2016, using the following terms: adherence, medication, medication management, medication list, and medication reminder. After identifying features within the retrieved apps, a final list of 42 features grouped into 4 modules (medication list, medication reminder, medication administration record, and additional features) was included in a questionnaire for expert consensus rating. A total of 52 experts in developmental disabilities, including persons with developmental disabilities, caregivers, and professionals, were invited to participate in a 3-round Delphi technique. The purpose was to obtain consensus on features that are preferred and suitable to promote independence in the medication management process among persons with developmental disabilities. Consensus for the first, second, and third rounds was defined as ≥90%, ≥80%, and ≥75% agreement, respectively.

**Results:**

A total of 75 responses were received over the 3 Delphi rounds—30 in the first round, 24 in the second round, and 21 in the third round. At the end of the third round, cumulative consensus was achieved for 60% (12/20) items in the medication list module, 100% (3/3) in the medication reminder module, 67% (2/3) in the medication administration record module, and 63% (10/16) in the additional features module. In addition to the medication list, medication reminder, and medication administration record features, experts selected the following top 3 most important additional features: automatic refills through pharmacies; ability to share medication information from the app with providers; and ability to share medication information from the app with family, friends, and caregivers. The top 3 least important features included a link to an official drug information source, privacy settings and password protection, and prescription refill reminders.

**Conclusions:**

Although several mobile apps for medication management exist, few are specifically designed to support persons with developmental disabilities in the complex medication management process. Of the 42 different features assessed, 64% (27/42) achieved consensus for inclusion in a future medication management app. This study provides information on the features of a medication management app that are most important to persons with developmental disabilities, caregivers, and professionals.

## Introduction

### Background

Developmental disabilities (DDs) is a term encompassing a range of disorders that are usually present at birth, last throughout the life span, and negatively affect the course of development of an individual at physical, intellectual, or emotional levels [1]. In the United States, the prevalence of any DD among children aged 3 to 17 years increased from 5.8% to 7.0% between 2014 to 2016 [2]. With improved life expectancy [3], adolescents and adults who have DDs naturally develop several chronic comorbidities over time [4], in addition to those intrinsically associated with their disability. People who have DDs are 2.5 times more likely to develop health problems, particularly neurological and psychological, compared with those who do not have a disability [5]. The increased risk for health problems has been verified from a young age, with adolescents who have disabilities displaying an increased risk to develop cardio-metabolic syndrome, cardiovascular disease, osteoporosis, and malignant diseases compared with their counterparts without a disability [6].

As a result of a high burden of diseases [7-9], polypharmacy, whose definition may vary across studies but most frequently denotes the use of 5 or more medications [10], is frequent among this population [11-14] and nonadherence can be an issue [15]. A study in primary care in the Netherlands revealed that 75% of people with DDs received medication compared with 59% individuals in the matched control group [16]. Data from Australia showed that polypharmacy (use of 5 or more medications) was highly prevalent and increased with age, affecting over 50% of individuals with a disability in the age group of 40 to 59 years [14]. Psychotropic medications are the most commonly prescribed class of drugs with reported use of over one-third of subjects enrolled in 2 different studies [5,13]. Despite high health care needs in this population, significant disparities in health and medical care utilization have consistently been reported [17,18].

Persons with DDs may live with their family (71%) or in a group home (13%) and often rely on caregivers for the performance of instrumental activities of daily living [19]. One such activity is medication management which involves several steps, including obtaining prescriptions from a physician, acquiring medications from a pharmacy, storing/taking/administering medications, monitoring for adverse and intended effects, and refilling medications [20]. Caregivers, with a range of medical knowledge and education levels, are usually required to be involved in each step of the medication use process [21], often encountering challenges in this multistep process [20]. Over the last decade, more emphasis has been placed on increasing the autonomy of people who have DDs, but the literature suggests that there is still considerable work to be done [22].

Technology is on the rise as a tool to promote independence in many areas including mobility, hearing and vision, communication, independent living, and computer use [23]. Existing and new technology can be used to empower persons with DDs to manage their own health issues during the transition to young adulthood [24]. The use of smartphone apps can be one strategy to support young adults who are transitioning to a state of independence to manage medications on their own. A 2016 survey assessing smartphone use among persons with DDs revealed that little difference in ownership rates between this population and the general population existed and that the use of smartphones for text messaging, emailing, using the Internet and social media, and using mobile apps increased among both groups [25].

### Medication Management Apps

At present, there are hundreds of medication management apps in the market, some designed to manage all medications [26] and others targeting specific disease states [27-32]. Medication management apps in the first group most often comprise basic features including keeping a medication list and sending reminders for medication taking, and less frequently, more advanced features such as the ability to track missed doses, exporting data for provider analysis, refill reminders, or managing multiple patient profiles [26,33]. Apps in the second group usually include features to monitor specific self-management activities or clinical parameters that are characteristic of different disease states [27]. Even though the evidence linking the use of apps to outcomes is sparse, 1 study showed that the use of an app positively impacted glycemic control and self-management in patients with diabetes [34].

With regard to apps specifically targeting persons with a DD, Dicianno and colleagues [35-37] developed an integrated system to support self-management of several health-related activities among individuals with spina bifida [38], which showed improvements in self-management abilities [35]. The interactive Mobile Health and Rehabilitation (iMHere) system comprises 6 smartphone modules providing reminders to individuals to take their medications, perform their catheterization and bowel management programs, examine their skin, complete a survey assessing depressive symptoms, and send and receive messages to and from the clinician. The medications (MyMeds) module, in addition to sending reminders, includes a list of all the medications taken by the user along with a photo and description, tracks whether medications were taken in response to reminders sent, and includes the pharmacy contact information to facilitate refill requests [38]. The iMHere system has 2-way communication between patients and providers and allows providers to remotely monitor adherence or address any problems related with medication management via a secure portal based on information provided by the user. Patient-centered technologic support such as iMHere is critical to support persons with DDs in every step of the medication management process.

### Objectives

Previous assessments of medication management apps highlighted their low quality [31,33,39] and showed that not a single app combines all the desirable features [33]. Before initiating any app development process, input from stakeholders including persons with DDs, caregivers, and professionals regarding the most preferred features for this population should be obtained. Therefore, the aim of this study was to identify medication management app features that are suitable to promote independence in the medication management process by young adults who have DDs using a Delphi consensus method.

## Methods

### Development of the Initial Questionnaire

**Figure 1:**
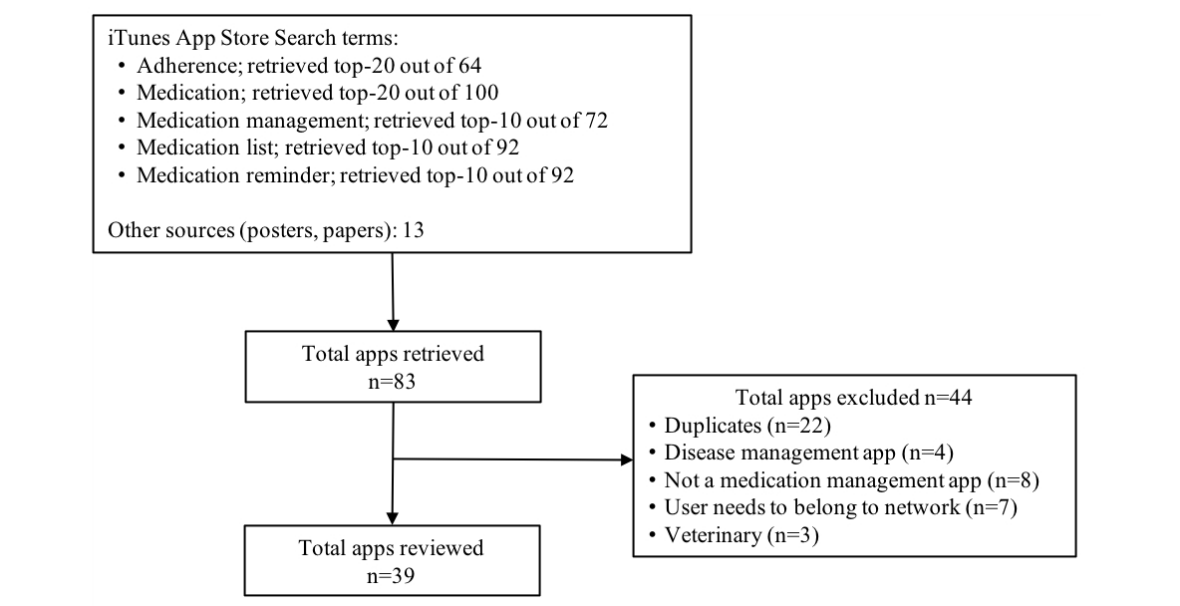
Flowchart of app search strategy and retrieval.

To identify medication management app features to be included in the Delphi questionnaire, a sample of medication management apps was reviewed. The works by Haase et al [40], Dinh et al [41], and Heldenbrand et al [42] served as a starting point to identify medication management app features. Furthermore, an apps search was conducted in Apple’s iTunes App Store, United States, in February 2016 to identify other relevant features using the following terms: adherence, medication, medication management, medication list, and medication reminder. Duplicate apps identified from the different search strategies were eliminated. Apps were included if they had any of the following features: medication list, dose reminders, dose documentation, or refill-assistance mechanisms. Apps designed for disease management but that did not include any features related with the medication management process were excluded. A subset of apps of each search strategy employed was reviewed, with a total of 39 apps ultimately being reviewed, to identify a list of app features (Figure 1).

Features of the apps reviewed were identified and summarized by 2 independent researchers (AF and TMS) based on the description included in the App Store and personal experience with the app after downloading and exploring its content and features. The common features identified across the apps, and that served as the basis for the questionnaire development, included the following: medication list, medication reminder, and medication administration report (including recording of medication taking). Additional features identified across the apps included prescription refill reminders; automatic pharmacy refill mechanisms; barcode scan to enter medications; use of a drug directory to enter new medications; ability to take a picture of the pill being added; indication of missed doses; overdose warnings; link to an official drug information source providing information about the disease and the medication; ability to export a file with medication taking data; record of vital signs home monitoring (eg, blood pressure, blood glucose); record of side effects; record of drug allergies; drug interaction checker; scan health documents and upload them to the app; ability to share medication information through the app (family, caregiver, health care provider); notification of caregiver if drug not taken; emergency contacts list; doctor name; doctor appointment reminder; pharmacy name; pharmacy locator; and gamification with rewards for users who adhere to their medication.

After identifying the list of medication app features, the research team evaluated and weighed the inclusion of each of these features in the final questionnaire, as well as the flow, organization, and format of the questions. The final version of the questionnaire (Multimedia Appendix 1) was organized into 4 modules that included (1) the medication list, (2) the medication reminder, (3) the medication administration record, and (4) additional features. Screenshots were selected from existing apps and chosen to illustrate different information presentation formats in the medication list, medication reminder, and medication administration record features. Experts were asked to rank 4 different screenshots per module, from most preferred to least preferred, based on design and visual appeal of the medication list, medication reminder, and medication administration record features. The answer format was a drag-and-drop list. Experts were then asked to rank the importance or essentiality of various specific features within the medication list, the medication reminder, and the medication administration record on a Likert scale (not important or not essential, less important or less essential, important or essential, and very important or very essential). For example, experts were asked to rank the importance of including specific medication, prescription, and pharmacy and prescriber information in the medication list feature. Module 4 presented a list of additional features identified from the reviewed apps and asked experts to rank their importance or essentiality on the same Likert scale. Furthermore, participants were asked to select the 3 most important and 3 least important additional features for inclusion in an app specifically designed for this population. The final portion of each of the 4 modules asked experts to provide comments on the features presented or suggest additional features not included in the initial questionnaire (free text response).

Before recruitment was initiated, the questionnaire was piloted with 3 individuals not directly associated with the research who provided feedback about the clarity of the wording and instructions, the answering format, and the flow of the questions in a *think aloud* fashion. The survey took about 15 min to complete.

### Selection and Recruitment of the Delphi Panel

A 3-round Delphi technique was used to gather consensus about medication management app features that would be suitable to assist young adults with DDs to independently manage their medications. The Delphi method utilizes a panel of experts in a field to establish consensus regarding a certain topic, with the ideal number of experts being between 15 and 30 [43]. In this study, a convenience sample of experts including persons with a DD, caregivers, researchers, and professionals who were familiar with the challenges and needs of persons with DDs was gathered to provide their insight on the best features to include in a future app designed specifically for this population and their caregivers. Experts were recruited from 2 sources: (1) the United Cerebral Palsy of Detroit Assistive Technology conference that took place in Detroit (MI) in May 2016 (n=25) and (2) the Technology Increasing knowledge: Technology Optimizing Choice (TIKTOC) Rehabilitation Engineering Research Center (RERC) Advisory Board and their mailing list (n=27). TIKTOC is a collaboration of clinicians and researchers at the University of Michigan whose mission is to develop and implement technology to optimize and support the self-management of personal health care and independence of individuals with physical, cognitive, and neuro DDs. Attendees of the United Cerebral Palsy of Detroit Assistive Technology conference provided their names and email addresses voluntarily to be part of the Delphi panel.

Experts were invited via an email, which included a link to the anonymous questionnaire on Qualtrics. The invitation encouraged experts to participate in all 3 rounds; however, they were not excluded from participating in a round if they missed the previous round(s). Participants were also able to make comments and suggest additional features which were reviewed by the research team at the end of each round, with relevant items being included in subsequent rounds. Each Delphi round lasted 1 week and, in-between rounds, we allowed 1 week before rolling out the following round to minimize respondent fatigue. The first round occurred between July 14 to 22, 2016; the second between July 28 to Aug 4, 2016; and the third between Aug 12 to 19, 2016. E-mail reminders were sent 3 days before the closing of each round to increase response rates. The process ensured anonymity of respondents at each round, and the number of individuals responding per round was noted. This study was approved as exempt by the University of Michigan Institutional Review Board (HUM00113908).

### Consensus Criteria and Data Analyses

Consensus criteria were established based on a progressive filtering process, with higher consensus requirements for the first Delphi round and progressively lower consensus for subsequent rounds—≥90%, ≥80%, and ≥75% agreement for the first, second, and third rounds, respectively. Items achieving consensus in each round were removed from the following rounds and not scored again.

For the screenshot ranking-type questions, consensus was calculated by assigning each screenshot a scaled score based on whether it was chosen as the first, second, third, or fourth option. Specifically, for a given screenshot option, we multiplied the number of experts by 1, 2, 3, or 4 (1=most preferred, 4=least preferred) according to how screenshots were ranked, creating a score for each screenshot. Lower scores indicated higher preference for a screenshot based on design and visual appeal.

For the Likert scale-type questions, consensus was calculated based on the percentage of positive versus negative responses for each feature, with positive being the sum of the percentage of experts that selected both important and very important or essential and very essential and negative being the sum of the percentage of experts that selected both not important and less important or not essential and less essential.

Descriptive statistics (percentages) were calculated to rate consensus obtained for each feature in the 3 Delphi rounds and to determine the 3 most and 3 least important additional features that should and should not, respectively, be included in a future app.

## Results

A total of 75 responses were received over the 3 Delphi rounds—30 in the first, 24 in the second, and 21 in the third round. Similar proportions of persons with disabilities and caregivers participated in the study, but most respondents were either researchers or professionals with an interest in DDs. Most respondents were female, with extensive experience and knowledge regarding DDs. Additional respondent characteristics are outlined in Table 1.

**Table 1:** Characteristics of respondents of each of the 3 Delphi rounds.

Demographic characteristic		Round 1 (n=30)	Round 2 (n=24)	Round 3 (n=21)
**Age (years)**				
	Mean (SD)	52.6 (17.1)	49.1 (18.8)	53.4 (19.6)
	Missing responses, n	10	7	9
**Gender**				
	Female, n (%)	13 (59)	9 (50)	10 (77)
	Missing responses, n	8	6	8
**Role^a^**				
	Person with a disability, n (%)	4 (17)	3 (16)	1 (7)
	Family caregiver for a person with a disability, n (%)	4 (17)	3 (16)	0
	Researcher with an interest in DDs^b^, n (%)	10 (44)	8 (42)	6 (43)
	Professional with an interest in DDs, n (%)	13 (57)	11 (58)	8 (57)
	Other, n (%)	2 (9)	1 (5)	1 (7)
	Missing responses, n	7	5	7
**Level of experience and knowledge regarding persons with disabilities**				
	Extensive, n (%)	14 (61)	11 (58)	8 (57)
	Some, n (%)	8 (35)	6 (32)	3 (21)
	Limited, n (%)	1 (4.3)	2 (11)	3 (21)
	Missing responses, n	7	5	7
**Disabilities individuals had direct experience with^a^**				
	Autism spectrum disorders, n (%)	12 (55)	11 (65)	6 (46)
	Cerebral palsy, n (%)	16 (73)	12 (71)	8 (62)
	Down syndrome, n (%)	13 (60)	11 (65)	5 (39)
	Fetal alcohol syndrome, n (%)	8 (36)	5 (29)	2 (15)
	Fragile X, n (%)	5 (23)	4 (24)	2 (15)
	Prader-Willi syndrome, n (%)	8 (36)	2 (12)	2 (15)
	Spina bifida	10 (46)	6 (35)	5 (39)
	Spinal cord injury, n (%)	14 (64)	14 (82)	9 (69)
	Williams syndrome, n (%)	6 (27)	3 (18)	2 (15)
	Other^c^, n (%)	11 (50)	6 (35)	3 (23)
	Missing responses, n	8	7	8

^a^Sum is >100% because participants selected more than 1 option.

^b^DDs: developmental disabilities.

^c^Includes amyotrophic lateral sclerosis, brachial plexus injury, brain injury (traumatic and acquired), Charcot-Marie-Tooth disease, dyslexia, mechanically ventilated patients, muscular dystrophy, other cognitive impairments, other genetic syndromes, Parkinson disease, and sensory-learning-injury.

### Module 1: Medication List

**Figure 2:**
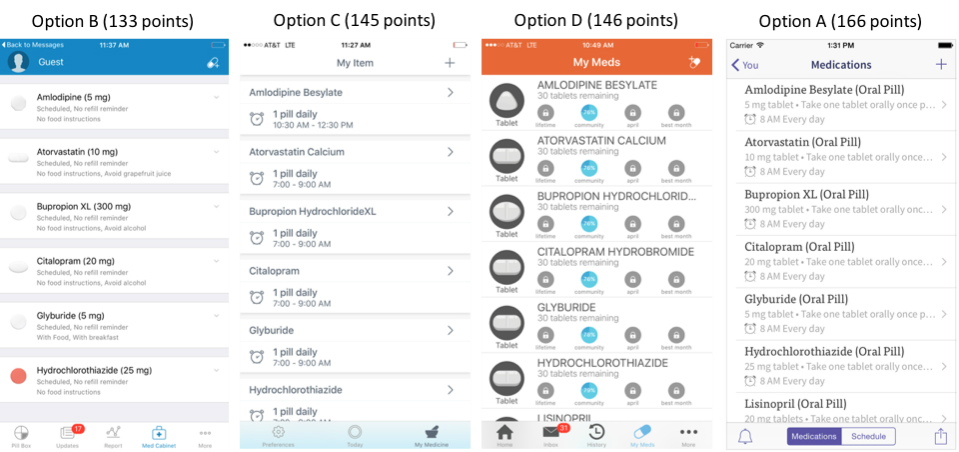
Medication list screenshots ranked from most preferred (lower score) to least preferred (higher score) based on design and visual appeal and respective scaled scores.

Of the 4 screenshots presenting different designs for the medication list, option B (133 points) was the most preferred among experts, but no consensus was achieved as to the best way to present the information. The distribution of experts selecting options C (145 points), D (146 points), and A (166 points) was very similar (Figure 2).

Of the 20 items assessed to be incorporated in the medication list feature of a medication management app, consensus was achieved for 12 (60%) throughout the 3 rounds (Multimedia Appendix 2). In the first round, consensus (≥90% agreement) was reached for the inclusion of instructions on how to take the medicine and quantity of the medicine to be taken. Round 2 resulted in consensus (≥80% agreement) on dosage, indication, number of prescription refills remaining, pharmacy phone number, drug directory to help populate medicines information, and scanning feature to enter medications on the list. In the third round, brand drug name, inclusion of a picture of the pill, name of the prescribing physician, and ability to upload the medication list from the pharmacy records were the items that achieved consensus (≥75% agreement; Multimedia Appendix 2).

Items suggested by participants that were included in further rounds were as follows: ability to upload a medication list from pharmacy records, which achieved consensus in round 3, and a speech-to-text feature to produce a medication list that did not achieve consensus as an essential feature.

### Module 2: Medication Reminder

**Figure 3:**
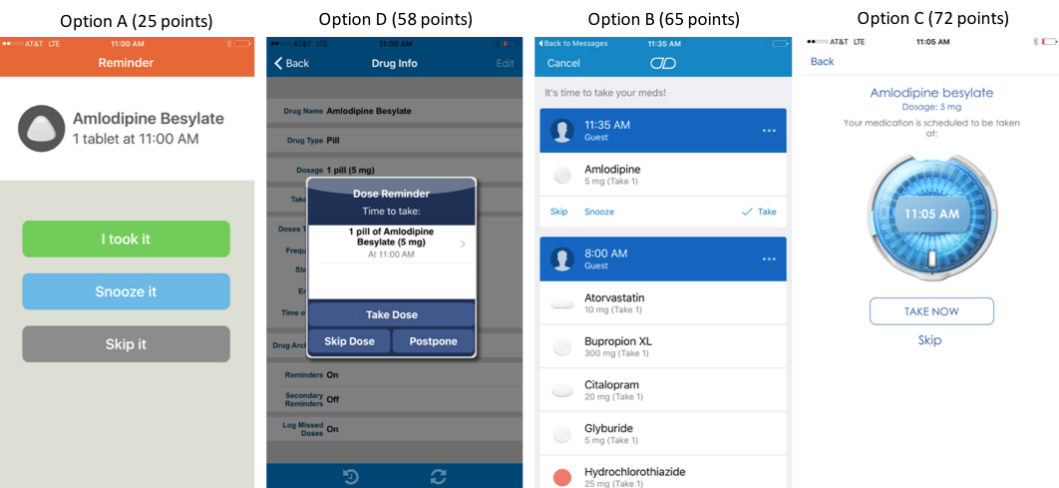
Medication reminder screenshots ranked from most preferred (lower score) to least preferred (higher score) based on design and visual appeal and respective scaled scores.

The design and visual appeal of option A (25 points) reached consensus among experts as the best to display medication taking reminders (Figure 3). All 3 items achieved consensus in the first round with experts agreeing that the medication reminder feature should include the options to report that the medication was taken, skipped, or postponed to be taken at another time (Multimedia Appendix 2).

### Module 3: Medication Administration Record

**Figure 4:**
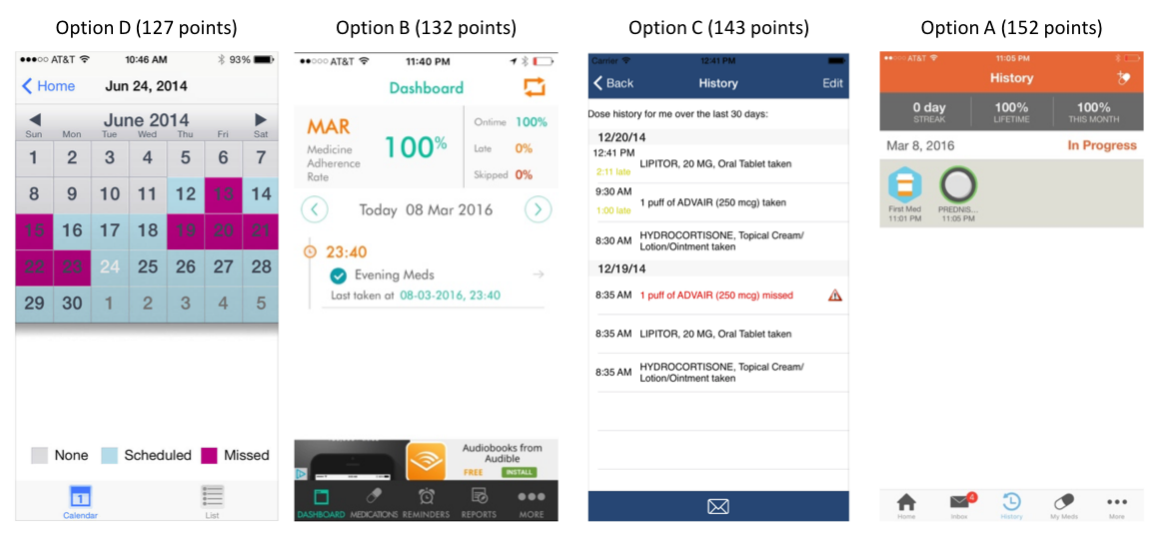
Medication Administration Record screenshots ranked from most preferred (lower score) to least preferred (higher score) based on design and visual appeal and respective scaled scores.

No consensus was obtained among experts as to the best design to present the record of medication administration, but the calendar format in option D (127 points) was preferred by most respondents, followed by options B (132 points), C (143 points), and A (152 points; Figure 4).

The report format showing days missed on a monthly calendar was the only one that achieved consensus in the first round. The report format showing days missed on a daily calendar also achieved consensus in the second round, but experts did not agree that showing the percent of doses taken would be helpful for persons with DDs to track medication taking (Multimedia Appendix 2).

### Module 4: Additional Features

A total of 63% (10/16) of additional features assessed reached consensus for inclusion in a medication management app. In the first round, experts agreed that the following features should be part of the app: ability to record known drug allergies, ability to share medication information from the app with providers and caregivers, emergency contact list, and prescription refill reminders (Multimedia Appendix 2). In the second round, experts agreed that ability to record side effects experienced, a drug interactions checker, and an overdose warning for maximum daily dose of *as needed* medication should be incorporated in a future app. In the third round, 2 other additional features reached consensus as highly important: automatic refill through pharmacies and doctor appointment reminders through the app. On the basis of expert feedback from previous rounds, 2 more features were included for assessment in the third round, but none achieved consensus: gaming system with rewards for adherent patients and ability to connect with peers through the app to motivate and share experiences with the app.

Finally, the top 3 most important additional features elected by experts were as follows: automatic refills through pharmacies; ability to share medication information from the app with providers; and ability to share medication information from the app with family, friends, and caregivers. The top 3 least important features included a link to an official drug information source, privacy settings and password protection, and prescription refill reminders.

In total, at the end of the 3 rounds, 27 of the 42 (64%) features assessed achieved consensus (Multimedia Appendix 2). For 12 out of the 27 (44%) features, unanimous concordance existed among persons with a DD or caregivers and the entire panel of experts; for 9 items (33%), concordance was above 75%; and for 6 items (22%), concordance with the panel was below 68%. The latter included dosage, indication, a report that showed days missed on a daily calendar, record/log of side effects experienced, drug interactions checker, and a picture of the pill highlighting any markings it may have.

The summary of items achieving consensus, and respective percent agreement, in each of the 3 rounds is presented in Multimedia Appendix 3.

### Comments From Experts

Overall, comments reflected the importance of apps to be simple, easy to use, customizable, and not overbearing in the amount of information they contain. There should be a balance between including options for individuals to personalize the app to their needs and including only those most important features to achieve the goal of improved adherence and greater independence for young adults with DDs. Respondents suggested that the app included different information depending on whether the user was a caregiver or a person with a DD, with more complex information being available to the previous and simpler and supportive information for self-management provided to the latter. Additionally, the app should have a system to assess the severity of side effects, provide decision support on how to manage side effects, and follow-up for evaluation.

Within the medication list feature, experts emphasized the importance of pictorial representations of the medication taking times to address low health literacy and suggested that medications be sorted according to the time of the day. Several experts suggested that a picture of the pill be included for each of the medications on the list with the ability to select from all possible shapes, sizes, colors, and manufacturer name to avoid confusion. One expert voiced that the screenshots presented too much information and advocated that end users provide ultimate feedback. Using speech-to-text functionality to enter medications on the list was supported by some experts, but discouraged by others who raised concerns about problems with pronunciation and inaccuracy of the medication entered. The alternative way consisting of having the information autopopulate as the user types in the first few letters of the medication was seen as risky and prone to errors because of the variety of presentations available in the market. Finally, experts believed the app should allow ordering medications from the pharmacy.

With regard to the medication reminder feature, experts suggested that the reminder delivery mode (eg, audio, vibrate, flashing screen) vary depending on whether medications were optional or mandatory and that a sketch of the pill be shown on the reminder screen. Geotagging reminders to go off when the person is at home or at work was another idea. Following the reminder, the app should provide feedback as an audio verbal and as a sound when patients tap the *took* option. Experts discouraged the use of *skip* as one of the options, because they believed this word seemed to give permission to be nonadherent. Rather, it was suggested that *not taken* or other neutral word be used. This should be followed by a text message to notify the caregiver. Several experts commented that the *snooze* option should be coupled with a functionality of how long to snooze the reminder for.

As per the medication administration record feature, comments that the dashboard (daily, weekly, monthly) should depend on the number of medications taken daily (many medications, display daily; few medications, display weekly or monthly) were made. A display of how the user is doing in terms of adherence compared with other peers or a gaming reward system to serve as a motivational effect to promote adherence were also proposed. The ability to email or export an adherence report within the app was deemed helpful, as was the ability to record reasons for missing doses.

A compilation of all the expert remarks is presented in Multimedia Appendix 4.

## Discussion

### Principal Findings and Comparison With Prior Work

In this study, we reviewed existing medication management apps and identified app features that appeared particularly relevant in supporting independence of young adults who have DDs during the medication management process. Of the 42 different features assessed, almost 65% achieved consensus for inclusion in a future medication management app.

The strength of our study is that the panel of experts included persons with disabilities as well as other stakeholders involved in their care. The number of experts included was within the recommended range [43], and we deliberately used high consensus criteria to ensure that only features that were considered critical by experts were identified. The list of features gathered and assessed by experts was similar to those reported in other studies [33,44-46], lending support to the accuracy of the features selected.

Within the medication list feature, experts considered information on how to take the medication and specific aspects such as dosage and indication essential. It was also important for experts that the app included ways to facilitate the entry of a new medication. This is especially critical for individuals with dexterity or visual impairments, as these are more prone to problems in task completion [47]. Although some forms of adding a medication to the list, such as uploading a medication list directly from the pharmacy records, are difficult to operationalize, others including scanning the prescription bottles or including a drug directory that automatically populates several fields as the user types in the name of the medication are already found in some of the current medication management apps.

The medication reminder feature, which is ubiquitous among medication management apps, should have 3 reporting options in an easy-to-use screen—taken, not taken, or snooze. Previous studies assessing the experiences of app users reported their desire to keep apps as simple as possible to make processes easier [47], including elderly people [48]. Simplicity was a key aspect highlighted by experts. On a similar note, the preferred display format of medication doses taken and missed was a monthly calendar, with doses taken and missed appearing in different colors for easy visualization.

The top 3 most important additional features selected by experts were the ability to perform automatic refills through pharmacies, to share medication information with providers, and to share medication information with family, friends, and caregivers. Of all the apps reviewed, only 1—Medisafe—included the feature to share medication information with caregivers, which would be of great value for young adults transitioning to independence. Previous studies demonstrated that involvement of caregivers through an app gives them a sense of better being able to monitor and control their child’s condition [44]. This feature is called Medfriend and it allows caregivers to track in real-time whether individuals take their medications and to be notified if they miss it. This feature assumes that users are able to tap the screen button if the medication is taken or skipped. Depending on the type and level of disability, some individuals may not be able to accomplish this task. The Medisafe app was ranked number 1 in recent quality assessments [33,46]. Currently, a randomized control trial is under way evaluating the impact of Medisafe on blood pressure control and medication adherence in the general population [49].

Several safety-related features such as recording side effects and drug allergies, overdose warning, or checking drug interactions also achieved consensus among experts. Specifically with regard to the latter, a recent study demonstrated that most of the apps containing a drug interaction checker did not identify all possible interactions associated with a given medication and half were unable to detect drug-herbal medication interactions [50]. Therefore, the accuracy of this feature needs to be ensured before inclusion in any app.

Additional features that did not achieve consensus, but that were initially thought to be important for young adults with DDs, were gamification (earning rewards or prizes for being adherent) and socialization. Mixed opinions about gamification were also found in a previous study assessing preferences of users of apps for cystic fibrosis [51]. However, the same and other studies reported socialization as a desired feature for users to find support in peers with the same condition [51,52].

As implications for future app development, participants in this and other studies emphasized the need for customization options to meet individual preferences, needs, and goals [51]; this may be especially relevant for customization for persons with DD versus their caregivers. More complex informatics systems integrating electronic medical records and pharmacy records would allow more advanced features that support independence of these individuals in the medication management process, including Web-based symptom reporting, automatic prescription refills, or sharing the same medication list with all providers. Incorporating or linking smart technology such as electronic pill bottles to the app would provide additional invaluable information to caregivers and providers to deliver better care to their patients [24].

The methodology used in this study does not provide the depth of information necessary to fully design an accessible app for medication management in this population, but it is a first step toward achieving that goal and provides useful information to adapt existing apps. Of note, as a result of the variety of levels of impairment across different disabilities, apps designed to help in the medication use process may be more applicable to persons who have mild-to-moderate impairment of cognitive or physical function, but this aspect would warrant further research.

### Limitations

This study has limitations. First, we selected the screenshots that we thought were the most different from each other and included 4 of them in the questionnaire. Many others could have been selected, yielding different results. However, the screenshots selected allowed the extraction of key design aspects to take into account in future app development. Second, researchers and professionals with an interest in DDs comprised the largest proportion of the panel of experts, which could have affected the results. Third, we did not track individual participation in each round, and this may have prevented input on items previously removed due to consensus being achieved. Finally, 1 limitation with the Delphi method is that the opportunity for experts to elaborate on their choices and produce a dialogue around controversial topics does not exist. This may have been partly addressed by the inclusion of free text comments sections at the end of each module.

### Conclusions

Although many mobile apps for medication management exist, few are specifically designed to support persons with DDs in the complex medication management process. This study provides information on the features of a medication management app that are most important to persons with DDs and their caregivers that should be taken into account by researchers, developers, and designers who create apps specifically designed for this population. Two key findings from this study are that simplicity is key and that constant communication between the person with a DD, the caregiver, and the health care professional through the app is critical.

Future research will require that a medication management app specifically designed for persons with DDs be developed or an existing app be adapted to include the features identified in this study. Further assessment of the impact of using an app on independence, medication adherence, self-management behaviors, and health outcomes is critical to guide their future use, widespread adoption, and, ultimately, financial support by payers.

## Appendix

Multimedia Appendix 1 Final questionnaire used to obtain expert consensus.

Multimedia Appendix 2 Final items to include and not to include in an app for young adults with disabilities.

Multimedia Appendix 3 Items achieving consensus in each of the 3 Delphi rounds.

Multimedia Appendix 4 Expert comments provided at the end of each module of the questionnaire.

## Abbreviations

DD: developmental disability
iMHere: interactive Mobile Health and Rehabilitation
RERC: Rehabilitation Engineering Research Center
TIKTOC: Technology Increasing knowledge: Technology Optimizing Choice

## References

[1] CDC.

[2] Zablotsky B, Black LI, Blumberg SJ (2017). Estimated prevalence of children with diagnosed developmental disabilities in the United States, 2014–2016. NCHS Data Brief, no 291.

[3] Thomas R, Barnes M (2010). Life expectancy for people with disabilities. NeuroRehabilitation.

[4] de Winter CF, Bastiaanse LP, Hilgenkamp TI, Evenhuis HM, Echteld MA (2012). Cardiovascular risk factors (diabetes, hypertension, hypercholesterolemia and metabolic syndrome) in older people with intellectual disability: results of the HA-ID study. Res Dev Disabil.

[5] van Schrojenstein Lantman-De Valk HM, Metsemakers JF, Haveman MJ, Crebolder HF (2000). Health problems in people with intellectual disability in general practice: a comparative study. Fam Pract.

[6] Wallén EF, Müllersdorf M, Christensson K, Malm G, Ekblom O, Marcus C (2009). High prevalence of cardio-metabolic risk factors among adolescents with intellectual disability. Acta Paediatr.

[7] Baxter H, Lowe K, Houston H, Jones G, Felce D, Kerr M (2006). Previously unidentified morbidity in patients with intellectual disability. Br J Gen Pract.

[8] Sohler N, Lubetkin E, Levy J, Soghomonian C, Rimmerman A (2009). Factors associated with obesity and coronary heart disease in people with intellectual disabilities. Soc Work Health Care.

[9] van Schrojenstein Lantman-de Valk HM, Walsh PN (2008). Managing health problems in people with intellectual disabilities. BMJ.

[10] Masnoon N, Shakib S, Kalisch-Ellett L, Caughey GE (2017). What is polypharmacy? A systematic review of definitions. BMC Geriatr.

[11] Burd L, Williams M, Klug MG, Fjelstad K, Schimke A, Kerbeshian J (1997). Prevalence of psychotropic and anticonvulsant drug use among North Dakota group home residents. J Intellect Disabil Res.

[12] Doan T, Ware R, McPherson L, van Dooren K, Bain C, Carrington S, Einfeld S, Tonge B, Lennox N (2014). Psychotropic medication use in adolescents with intellectual disability living in the community. Pharmacoepidemiol Drug Saf.

[13] Doan TN, Lennox NG, Taylor-Gomez M, Ware RS (2013). Medication use among Australian adults with intellectual disability in primary healthcare settings: a cross-sectional study. J Intellect Dev Disabil.

[14] Department of Health & Human Services (2015). Victorian Population Health Survey of People with an Intellectual Disability 2013.

[15] Vacek JL, Hunt SL, Shireman T (2013). Hypertension medication use and adherence among adults with developmental disability. Disabil Health J.

[16] Straetmans JM, van Schrojenstein Lantman-de Valk HM, Schellevis FG, Dinant G (2007). Health problems of people with intellectual disabilities: the impact for general practice. Br J Gen Pract.

[17] Havercamp SM, Scandlin D, Roth M (2004). Health disparities among adults with developmental disabilities, adults with other disabilities, and adults not reporting disability in North Carolina. Public Health Rep.

[18] Emerson E, Hatton C, Baines S, Robertson J (2016). The physical health of British adults with intellectual disability: cross sectional study. Int J Equity Health.

[19] Braddock D, Hemp R, Rizzolo MC, Tanis ES, Haffer L, Wu J (2015). The State of the States in Intellectual and Developmental Disabilities: Emerging from the Great Recession. American Association of Intellectual and Developmental Disabilities, editor. 10th ed.

[20] Erickson SR, Salgado TM, Tan X (2016). Issues in the medication management process in people who have intellectual and developmental disabilities: a qualitative study of the caregivers' perspective. Intellect Dev Disabil.

[21] Erickson SR, LeRoy B (2015). Health literacy and medication administration performance by caregivers of adults with developmental disabilities. J Am Pharm Assoc (2003).

[22] Wullink M, Widdershoven G, van Schrojenstein Lantman-de Valk H, Metsemakers J, Dinant GJ (2009). Autonomy in relation to health among people with intellectual disability: a literature review. J Intellect Disabil Res.

[23] Palmer SB, Wehmeyer ML, Davies DK, Stock SE (2012). Family members' reports of the technology use of family members with intellectual and developmental disabilities. J Intellect Disabil Res.

[24] Haymes LK, Storey K, Maldonado A, Post M, Montgomery J (2015). Using applied behavior analysis and smart technology for meeting the health needs of individuals with intellectual disabilities. Dev Neurorehabil.

[25] Morris JT, Mark Sweatman W, Jones ML (2017). Smartphone use and activities by people with disabilities: user survey 2016. J Technol Pers Disabil.

[26] Dayer L, Heldenbrand S, Anderson P, Gubbins PO, Martin BC (2013). Smartphone medication adherence apps: potential benefits to patients and providers. J Am Pharm Assoc (2003).

[27] Brzan PP, Rotman E, Pajnkihar M, Klanjsek P (2016). Mobile applications for control and self management of diabetes: a systematic review. J Med Syst.

[28] Bardus M, van Beurden SB, Smith JR, Abraham C (2016). A review and content analysis of engagement, functionality, aesthetics, information quality, and change techniques in the most popular commercial apps for weight management. Int J Behav Nutr Phys Act.

[29] Sobnath DD, Philip N, Kayyali R, Nabhani-Gebara S, Pierscionek B, Vaes AW, Spruit MA, Kaimakamis E (2017). Features of a mobile support app for patients with chronic obstructive pulmonary disease: literature review and current applications. JMIR Mhealth Uhealth.

[30] Grainger R, Townsley H, White B, Langlotz T, Taylor WJ (2017). Apps for people with rheumatoid arthritis to monitor their disease activity: a review of apps for best practice and quality. JMIR Mhealth Uhealth.

[31] Nicholas J, Larsen ME, Proudfoot J, Christensen H (2015). Mobile apps for bipolar disorder: a systematic review of features and content quality. J Med Internet Res.

[32] Masterson Creber RM, Maurer MS, Reading M, Hiraldo G, Hickey KT, Iribarren S (2016). Review and analysis of existing mobile phone apps to support heart failure symptom monitoring and self-care management using the Mobile Application Rating Scale (MARS). JMIR Mhealth Uhealth.

[33] Santo K, Richtering SS, Chalmers J, Thiagalingam A, Chow CK, Redfern J (2016). Mobile phone apps to improve medication adherence: a systematic stepwise process to identify high-quality apps. JMIR Mhealth Uhealth.

[34] Liang X, Wang Q, Yang X, Cao J, Chen J, Mo X, Huang J, Wang L, Gu D (2011). Effect of mobile phone intervention for diabetes on glycaemic control: a meta-analysis. Diabet Med.

[35] Dicianno BE, Fairman AD, McCue M, Parmanto B, Yih E, McCoy A, Pramana G, Yu DX, McClelland J, Collins DM, Brienza DM (2016). Feasibility of using mobile health to promote self-management in spina bifida. Am J Phys Med Rehabil.

[36] Fairman AD, Dicianno BE, Datt N, Garver A, Parmanto B, McCue M (2013). Outcomes of clinicians, caregivers, family members and adults with spina bifida regarding receptivity to use of the iMHere mHealth solution to promote wellness. Int J Telerehabil.

[37] Yu DX, Parmanto B, Dicianno BE, Pramana G (2015). Accessibility of mHealth self-care apps for individuals with spina bifida. Perspect Health Inf Manag.

[38] Parmanto B, Pramana G, Yu DX, Fairman AD, Dicianno BE, McCue MP (2013). iMHere: a novel mHealth system for supporting self-care in management of complex and chronic conditions. JMIR Mhealth Uhealth.

[39] Bailey SC, Belter LT, Pandit AU, Carpenter DM, Carlos E, Wolf MS (2014). The availability, functionality, and quality of mobile applications supporting medication self-management. J Am Med Inform Assoc.

[40] Haase J, Farris KB, Dorsch MP (2016). Mobile applications to improve medication adherence. Telemed J E Health.

[41] Dinh T, Choy J, Lee K (2016). Mobile applications for medication self-management and adherence.

[42] Heldenbrand S, Martin B, Shilling R, VanValkenburg M, Renna C, Hadden K, Dayer L (2016). There are too many apps for that: development of a website helps patients and providers find quality medication adherence applications.

[43] Linstone H, Turoff M (2002). The Delphi Method: Techniques and Applications.

[44] Geryk LL, Roberts CA, Sage AJ, Coyne-Beasley T, Sleath BL, Carpenter DM (2016). Parent and clinician preferences for an asthma app to promote adolescent self-management: a formative study. JMIR Res Protoc.

[45] Mendiola MF, Kalnicki M, Lindenauer S (2015). Valuable features in mobile health apps for patients and consumers: content analysis of apps and user ratings. JMIR Mhealth Uhealth.

[46] Heldenbrand S, Martin BC, Gubbins PO, Hadden K, Renna C, Shilling R, Dayer L (2016). Assessment of medication adherence app features, functionality, and health literacy level and the creation of a searchable Web-based adherence app resource for health care professionals and patients. J Am Pharm Assoc (2003).

[47] Yu DX, Parmanto B, Dicianno BE, Watzlaf VJ, Seelman KD (2017). Accessibility needs and challenges of a mHealth system for patients with dexterity impairments. Disabil Rehabil Assist Technol.

[48] Grindrod KA, Li M, Gates A (2014). Evaluating user perceptions of mobile medication management applications with older adults: a usability study. JMIR Mhealth Uhealth.

[49] Morawski K, Ghazinouri R, Krumme A, McDonough J, Durfee E, Oley L, Mohta N, Juusola J, Choudhry NK (2017). Rationale and design of the Medication adherence Improvement Support App For Engagement-Blood Pressure (MedISAFE-BP) trial. Am Heart J.

[50] Loy JS, Ali EE, Yap KY (2016). Quality assessment of medical apps that target medication-related problems. J Manag Care Spec Pharm.

[51] Hilliard ME, Hahn A, Ridge AK, Eakin MN, Riekert KA (2014). User preferences and design recommendations for an mHealth app to promote cystic fibrosis self-management. JMIR Mhealth Uhealth.

[52] Milward J, Khadjesari Z, Fincham-Campbell S, Deluca P, Watson R, Drummond C (2016). User preferences for content, features, and style for an app to reduce harmful drinking in young adults: analysis of user feedback in app stores and focus group interviews. JMIR Mhealth Uhealth.

