# Supplementary material for: Identifying Medication Management Smartphone App Features Suitable for Young Adults With Developmental Disabilities: Delphi Consensus Study
Source: JMIR Mhealth Uhealth. 2018 May 23;6(5):e129. doi: 10.2196/mhealth.9527 (PMC5990856; doi:10.2196/mhealth.9527)
Supplement: Multimedia Appendix 1 [file mhealth_v6i5e129_app1.pdf]

Multimedia Appendix 1: Final questionnaire used to obtain expert consensus.

General Information:
You have been asked to participate in this questionnaire based on your expertise in the field of intellectual and developmental disabilities. Experts may include professionals, researchers, paid caregivers and family caregivers, as well as, individuals with intellectual and developmental disabilities.

A number of existing medication management smartphone applications (apps) have been reviewed and many features identified. The purpose of this questionnaire is to collect feedback on some of the main features identified.

This information will be used to identify the most important features to include in an app targeted for use by **individuals with intellectual and developmental disabilities and their caregivers** and the best way to present those features so they are well designed and visually appealing.

The aim of this project is to obtain expert feedback on:
1. The visual appeal and design of existing app features
2. The importance of various features included in some apps
3. Any additional feedback on features to be included in an app

Instructions:
The questionnaire will take 10 to 15 minutes to complete and is divided into four modules. Each module will ask you three types of questions. The first question will present you with different app screenshots and ask for your opinion regarding their visual appeal and design. The second question will ask you to rank the importance of various features. The third question will ask for any additional feedback.

Please consider each question keeping in mind the patient population we are targeting. We intend for the information gathered through this questionnaire to apply universally to individuals with intellectual and developmental disabilities and their caregivers.

University of Michigan
Consent to be part of a research study

Title of Project: Mobile Apps for Individuals with Intellectual and Developmental Disabilities
PharmD Candidate: Alexa Fedrigon
Faculty Advisors: Karen Farris, PhD, Teresa Salgado, PhD

Thank you for being part of this important project. Please know that:
- Participation in the questionnaire is completely voluntary
- You may skip answering any questions you wish
- You may stop the questionnaire at any time
- The answers you give are confidential
- Your answers will be used for research only
- Your answers will be securely stored on password protected UM servers

CONTACT INFORMATION:
If you have any questions about the questionnaire, you may email Alexa Fedrigon (fedrigon@med.umich.edu) or Teresa Salgado (tsalgado@med.umich.edu) and we will respond as soon as possible.

[Multiple Choice]
- Yes, I agree with the terms above
- No, I do not agree with the terms above

Module One: Medication List
The majority of medication management apps include a medication list feature which allows the user to document each of their medications and information about how they should be taken. The purpose of this module is to assess components of the medication list feature.

Rank the following example screen shots from most preferred to least preferred based on design and visual appeal. The four examples below are labeled A, B, C or D. Go to the bottom of this page and drag-and-drop the options into the order that you prefer, with your first choice on top and your last choice on bottom.

Option A

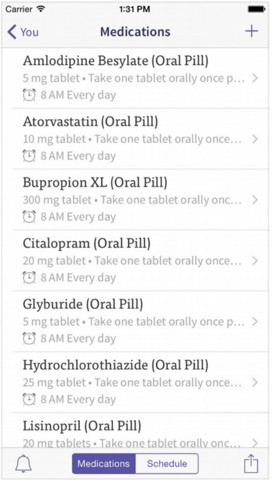

Option B

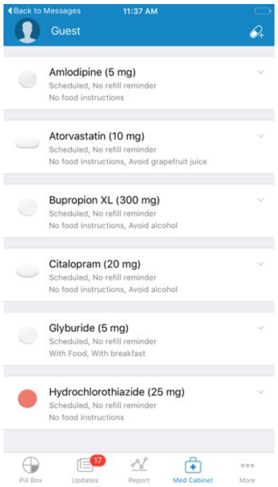

Option C

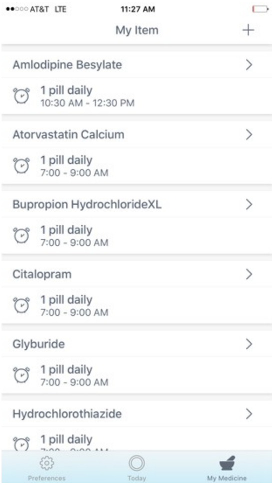

Option D

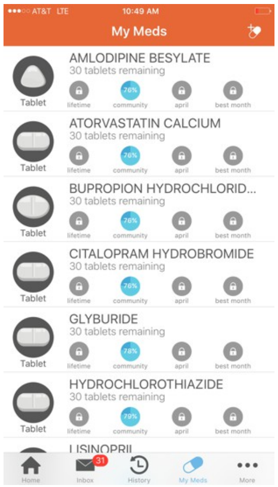

On a scale from “Not essential” to “Very essential”, please rate each medication information item based on whether you think it is essential when adding a new medication to the app:

- Generic drug name (atorvastatin, bupropion, citalopram, etc.)
- Brand drug name (Lipitor, Wellbutrin, Celexa, etc.)
- Dosage (20, 100, etc.)
- Units (mg, mcg, IU, etc.)
- Quantity (number of pills, etc.)
- Instructions (with food, before eating, on an empty stomach, time of day to be taken, etc.)
- What it is for or indication (pain, infection, high blood pressure, etc.)
- Inclusion of a picture of the pill highlighting any markings it may have

On a scale from “Not essential” to “Very essential”, please rate each prescription information item based on whether you think it is essential when adding a new medication to the app:

- Prescription number
- Prescription date
- Prescription refills remaining
- Prescription expiration date

On a scale from “Not essential” to “Very essential”, please rate each pharmacy and prescriber information item based on whether you think it is essential when adding a new medication to the app:

- Name of prescribing physician
- Physician’s office phone number
- Pharmacy name
- Pharmacy phone number

When adding a medication to the medication list there is a set of information about each medication that needs to be entered into the app. Often this information must be manually entered for each medication. However, there are two alternatives which help the user add new medications and enter the medication information to the medication list. These alternative features could be utilized by an individual entering their own medication list or by their caregiver.

The first alternative feature is a drug directory that helps populate all the data required in the medication list as the individual begins to type in a new medication.

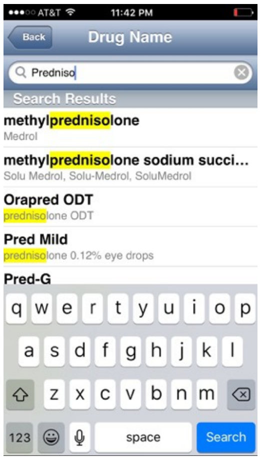

On a scale from “Not important” to “Very important”, please rate the <u>importance</u> of this feature in a medication management app:

- Inclusion of a drug directory that helps populate all the data required in the medication list as the individual adds a new medication.

The second alternative feature is the ability to scan prescription bottles using the phone's camera to create a medication list, rather than manually entering the information. This feature would allow the medication name, dose and instructions to be entered automatically rather than manually.

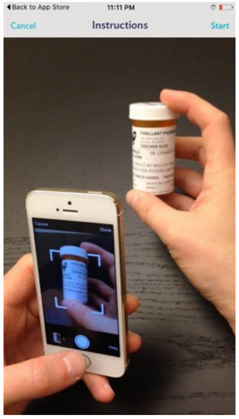

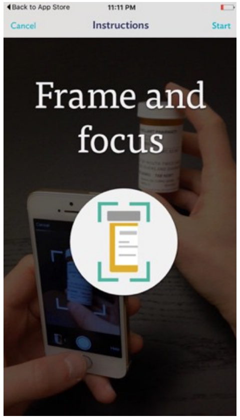

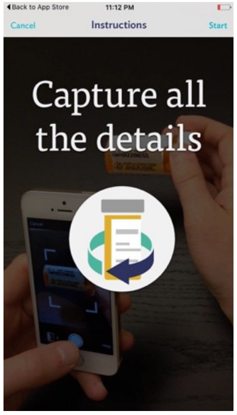

On a scale from "Not important" to "Very important", please rate the <u>importance</u> of including this feature in a medication management app:

- The ability to scan prescription bottles using the phone's camera to create a medication list, rather than manually entering the information.

Please include any other feature(s) that you would find helpful to be included in the <u>medication list</u> function.

- [Free text]

Module Two: Medication Reminder

The majority of medication management apps include a medication reminder feature which allows the user to program an alarm or reminder when they should take their medication. The purpose of this module is to assess components of the medication reminder feature.

Rank the following example screen shots from most preferred to least preferred based on <u>design and visual appeal. The four examples below are labeled A, B, C or D.</u> Go to the bottom of this page and drag-anddrop the options into the order that you prefer, with your first choice on top and your last choice on bottom.

Option A

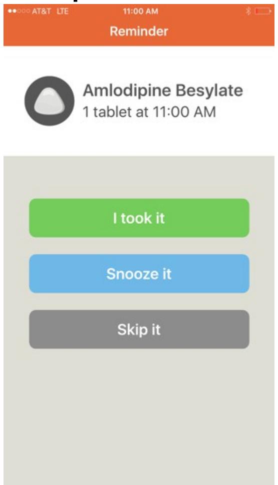

Option B

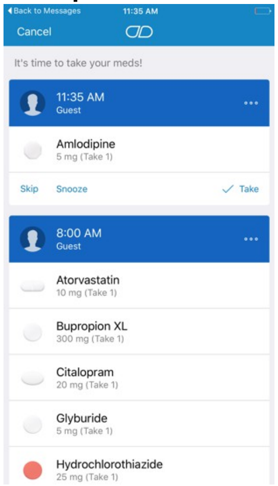

Option C

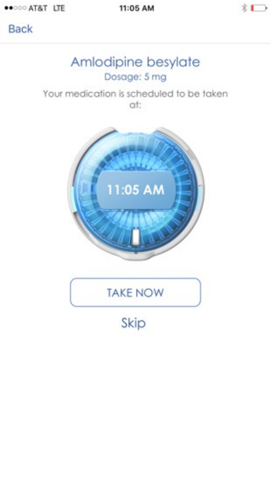

Option D

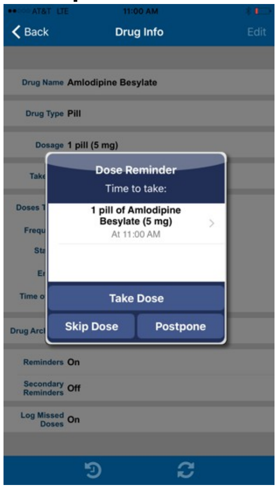

Some medication reminders allow the user to indicate that they took the medication, waited to take the medication later or skipped taking the medication at that time.

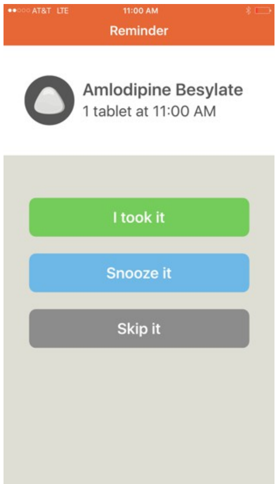

On a scale from “Not important” to “Very important”, please rate the <u>importance</u> of including the following features in a medication management app:

- An option to report medication taking after receiving the reminder or “take”.
- An option to delay the reminder so it notifies you later, “snooze” or “postpone”.
- An option to report that the medication was not taken after receiving the reminder or “skip”

Please include any other feature(s) that you would find helpful to be included in the <u>medication reminder</u> function.

- [Free text]

Module Three: Medication Administration Report

The majority of medication management apps include a medication administration report feature which includes information about how often, when and if an individual took their medication. The purpose of this module is to assess components of the <u>medication administration report</u> feature.

Rank the following example screen shots from most preferred to least preferred based on <u>design and visual appeal</u>. The four examples below are labeled A, B, C or D. Go to the bottom of this page and drag-and-drop the options into the order that you prefer, with your first choice on top and your last choice on bottom.

Option A

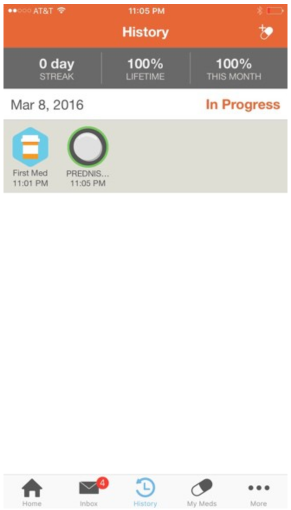

Option B

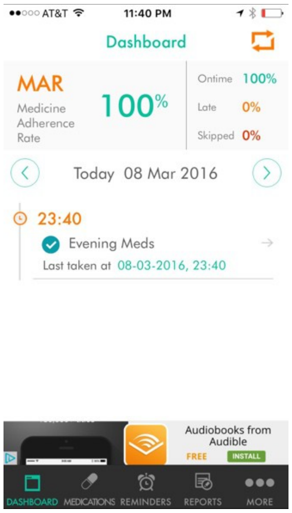

Option C

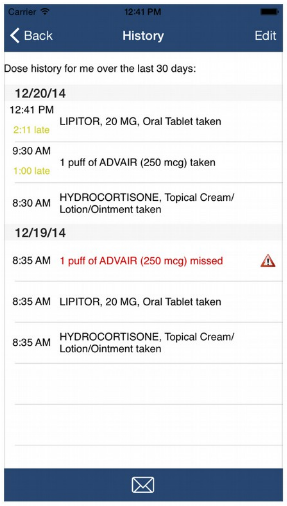

Option D

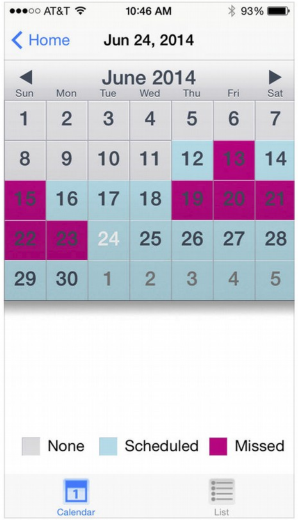

On a scale from “Not important” to “Very important”, please rate the importance of including the following features in a medication management app:

- A report that shows a percent of doses taken.
- A report that shows days missed on a daily calendar.
- A report that shows days missed on a monthly calendar.

Please include any other feature(s) that you would find helpful to be included in the medication administration report function.

- [Free text]

Module Four: Other Features

In reviewing the many available medication management apps, we can see that medication lists, medication reminders and medication administration reports are standard functions available in most medication management apps. However, many apps contain additional features related to drug information, information sharing and pharmacy information. This last module will ask you to consider how essential each of these features are and how likely you would be to use those features in the care of an individual with an intellectual or developmental disability.

On a scale from “Not essential” to “Very essential”, please rate the following features based on whether you consider each drug information feature important for caregiver and/or patient use in a medication management app.

- Record/log of side effects experienced
- Drug interactions checker
- Additional information about the drug (i.e. link to an official drug information source containing information about how the drug works, what side effects are associated with the drug, etc.)
- Overdose warning for maximum daily dose of as needed medications
- Record/log of known drug allergies
- Record/log for home monitoring of blood pressure, glucose levels, asthma control, spasticity level, etc.

On a scale from “Not essential” to “Very essential”, please rate the following features based on whether you consider each information sharing, storing or privacy feature important for caregiver and/or patient use in a medication management app.

- Ability to share medication information from the app with family, friends or caregiver
- Ability to share medication information from the app with provider (physician, pharmacist, nurse, etc.)
- Emergency contact list
- Privacy settings and password protection

On a scale from “Not essential” to “Very essential”, please rate the following features based on whether you consider each pharmacy information and reminders feature important for caregiver and/or patient use in a medication management app.

- Pharmacy locator function to find a pharmacy near you
- Automatic refill mechanism through pharmacy
- Prescription refill reminders
- Doctor appointment reminders

On a scale from "Not likely" to "Very likely", please rate the following based on whether each drug information feature is something you personally would utilize in the care of an individual with an intellectual or developmental disability.

- Record/log of side effects experienced
- Drug interactions checker
- Additional information about the drug (i.e. link to an official drug information source containing information about how the drug works, what side effects are associated with the drug, etc.)
- Overdose warning for maximum daily dose of as needed medications
- Record/log of known drug allergies
- Record/log for home monitoring of blood pressure, glucose levels, asthma control, spasticity level, etc.

On a scale from "Not likely" to "Very likely", please rate the following based on whether each information sharing, storing or privacy feature is something you personally would utilize in the care of an individual with an intellectual or developmental disability.

- Ability to share medication information from the app with family, friends or caregiver
- Ability to share medication information from the app with provider (physician, pharmacist, nurse, etc.)
- Emergency contact list
- Privacy settings and password protection

On a scale from "Not likely" to "Very likely", please rate the following based on whether each pharmacy information and reminders feature is something you personally would utilize in the care of an individual with an intellectual or developmental disability.

- Pharmacy locator function to find a pharmacy near you
- Automatic refill mechanism through pharmacy
- Prescription refill reminders
- Doctor appointment reminders

As indicated earlier, medication lists, medication reminders and medication administration reports are common features. Now, choose the top three <u>most important</u> **additional** features for inclusion in a medication management app.

Drag and drop three items from the left hand column to the box to make your selections.

- Record/log of side effects experienced
- Drug interactions checker
- Additional information about the drug (i.e. link to an official drug information source containing information about how the drug works, what side effects are associated with the drug, etc.)
- Overdose warning for maximum daily dose of as needed medications
- Record/log of known drug allergies
- Record/log for home monitoring of blood pressure, glucose levels, asthma control, spasticity level, etc.
- Ability to share medication information from the app with family, friends or caregiver
- Ability to share medication information from the app with provider
- Emergency contact list
- Privacy settings and password protectionPharmacy locator function to find a pharmacy near you
- Automatic refill mechanism through pharmacy
- Prescription refill reminders
- Doctor appointment reminders

As indicated earlier, medication lists, medication reminders and medication administration reports are common features. Now, choose the three <u>least important</u> **additional** features for inclusion in a medication management app.

Drag and drop three items from the left hand column to the box to make your selections.

- Record/log of side effects experienced
- Drug interactions checker
- Additional information about the drug (i.e. link to an official drug information source containing information about how the drug works, what side effects are associated with the drug, etc.)
- Overdose warning for maximum daily dose of as needed medications
- Record/log of known drug allergies
- Record/log for home monitoring of blood pressure, glucose levels, asthma control, spasticity level, etc.
- Ability to share medication information from the app with family, friends or caregiver
- Ability to share medication information from the app with provider
- Emergency contact list
- Privacy settings and password protection
- Pharmacy locator function to find a pharmacy near you
- Automatic refill mechanism through pharmacy
- Prescription refill reminders
- Doctor appointment reminders

Please include any other feature that you would find helpful to be included in a <u>medication management app</u>.

- [Free text]

Demographics
Lastly, our research team is interested in collecting additional information describing who has participated in our survey. Please answer the following questions about yourself.

Please select which best describes you.
- I am an individual with a disability
- I am a family caregiver for an individual with a disability
- I am a paid caregiver for an individual with a disability
- I am a researcher with an interest in individuals with disabilities
- I am a professional with an interest in individuals with disabilities
- Other [free text]

Please select the option describing your level of experience and knowledge regarding individuals with disabilities.
- Extensive
- Some
- Limited
- None

Please indicate which disabilities you have direct experience with. You may select more than one option below.
- Autism Spectrum Disorders
- Cerebral palsy
- Down syndrome
- Fetal alcohol syndrome
- Fragile X
- Prader Willi Syndrome
- Spina Bifida
- Spinal cord injury
- Williams Syndrome
- Other [free text]

What is your age?
- [free response]

What is your sex?
- Male
- Female
