# Supplementary material for: Identifying Medication Management Smartphone App Features Suitable for Young Adults With Developmental Disabilities: Delphi Consensus Study
Source: JMIR Mhealth Uhealth. 2018 May 23;6(5):e129. doi: 10.2196/mhealth.9527 (PMC5990856; doi:10.2196/mhealth.9527)
Supplement: Multimedia Appendix 2 [file mhealth_v6i5e129_app2.pdf]

Multimedia Appendix 2: Features achieving and not achieving consensus to be included in a medication management app designed for persons with developmental disabilities (DDs) over the 3 Delphi rounds.

App Features	Features achieving consensus	Features not achieving consensus
**Module 1: Medication list—medication information**		
	Brand drug name (Lipitor, Wellbutrin, Celexa, etc)	Generic drug name (atorvastatin, bupropion, citalopram, etc)
	Dosage (20, 100, etc)	Units (mg, mcg, IU, etc)
	Quantity (number of pills, etc)	
	Instructions (with food, before eating, on an empty stomach, time of day to be taken, etc)	
	What it is for or indication (pain, infection, high blood pressure, etc)	
	Inclusion of a picture of the pill highlighting any markings it may have	
**Module 1: Medication list—prescription information**		
	Prescription refills remaining	Prescription number
		Prescription date
		Prescription expiration date
**Module 1: Medication list—pharmacy and prescriber information**		
	Name of prescribing physician	Physician’s office phone number
	Pharmacy phone number	Pharmacy name
**Module 1: Medication list—alternative features**		
	Inclusion of a drug directory that helps populate all the data required in the medication list as the individual adds a new medication	Speech-to-text technology that helps populate all the data required in the medication list as the individual verbally relays information about the new medication
	The ability to scan prescription bottles using the phone’s camera to create a medication list, rather than manually entering the information	
	Ability to upload a medication list directly from the pharmacy records	
**Module 2: Medication reminder**		

	An option to report medication taking after receiving the reminder or *take*	
	An option to delay the reminder so it notifies you later, *snooze* or *postpone*	
	An option to report that the medication was not taken after receiving the reminder or *skip*	
**Module 3: Medication administration report**		
	A report that shows days missed on a monthly calendar	A report that shows percentage of the doses taken
	A report that shows days missed on a daily calendar	
**Module 4: Additional features—drug information**		
	Record or log of side effects experienced	Additional information about the drug (ie, link to an official drug information source containing information about how the drug works, what side effects are associated with the drug, etc)
	Drug interactions checker	Record/log for home monitoring of blood pressure, glucose levels, asthma control, spasticity level, etc
	Overdose warning for maximum daily dose of as needed medications	
	Record/log of known drug allergies	
**Module 4: Additional features—information sharing, storing, or privacy**		
	Ability to share medication information from the app with family, friends, or the caregiver	Privacy settings and password protection
	Ability to share medication information from the app with provider (physician, pharmacist, nurse, etc)	
	Emergency contact list	
**Module 4: Additional features—pharmacy information and reminders**		
	Automatic refill mechanism through your pharmacy	Pharmacy locator function to find a pharmacy near you
	Prescription refill reminders	
	Doctor appointment reminders	
**Module 4: Additional features—other**		

		Inclusion of a gaming system with points or rewards for successful utilization
		Ability to connect with peers through the app to motivate and share adherence
