# Supplementary material for: Identifying Medication Management Smartphone App Features Suitable for Young Adults With Developmental Disabilities: Delphi Consensus Study
Source: JMIR Mhealth Uhealth. 2018 May 23;6(5):e129. doi: 10.2196/mhealth.9527 (PMC5990856; doi:10.2196/mhealth.9527)
Supplement: Multimedia Appendix 3 [file mhealth_v6i5e129_app3.pdf]

**Multimedia Appendix 3:** Items achieving consensus in each of the 3 Delphi rounds.

Percentage of experts ranking each item as essential (+), non-essential (-), important (+) or non-important (-) features during the three Delphi rounds. The colors reflect consensus achieved for items considered essential or important features (green) or non-essential or non-important features (red). The gray shade means that items were not assessed and white cells mean that consensus was not attained among experts.

| App Feature Item Description | Delphi Round | | |
|---|---|---|---|
| | R1 (≥90%) | R2 (≥80%) | R3 (≥75%) |
| **MODULE 1** | | | |
| **Medication List – Medication Information** | | | |
| Generic drug name (atorvastatin, bupropion, citalopram, etc.) | 75% (+) | 63.2% (+) | 68.8% (+) |
| Brand drug name (Lipitor, Wellbutrin, Celexa, etc.) | 75% (+) | 73.7% (+) | 75% (+) |
| Dosage (20, 100, etc.) | 83.3% (+) | 84.2% (+) | |
| Units (mg, mcg, IU, etc.) | 58.3% (+) | 63.2% (+) | 62.5% (+) |
| Quantity (number of pills, etc.) | 100% (+) | | |
| Instructions (with food, before eating, on an empty stomach, time of day to be taken, etc.) | 91.7% (+) | | |
| What it is for or indication (pain, infection, high blood pressure, etc.) | 83.3% (+) | 84.2% (+) | |
| Inclusion of a picture of the pill highlighting any markings it may have | 83.3% (+) | 73.7% (+) | 87.5% (+) |
| **Medication List – Prescription Information** | | | |
| Prescription number | 58.3% (-) | 60% (+) | 56.3% (+) |
| Prescription date | 58.3% (-) | 57.9% (+) | 62.5% (+) |
| Prescription refills remaining | 83.3% (+) | 95% (+) | |
| Prescription expiration date | 79.2% (+) | 75% (+) | 62.5% (+) |
| **Medication List – Pharmacy and Prescriber Information** | | | |
| Name of prescribing physician | 66.7% (+) | 65% (+) | 81.3% (+) |
| Physician's office phone number | 75% (+) | 55% (+) | 53.3% (+) |
| Pharmacy name | 66.7% (+) | 75% (+) | 68.8% (+) |
| Pharmacy phone number | 58.3% (+) | 95% (+) | |
| **Medication List - Alternative features** | | | |
| Inclusion of a drug directory that helps populate all the data required in the medication list as the individual adds a new medication | 78.3% (+) | 90.5% (+) | |
| The ability to scan prescription bottles using the phone's camera to create a medication list, rather than manually entering the information | 83.3% (+) | 85.7% (+) | |
| Speech-to-text technology that helps populate all the data required in the medication list as the individual verbally relays information about the new medication | | | 56.3% (+) |
| Ability to upload a medication list directly from the pharmacy records | | | 93.3% (+) |
| **MODULE 2** | | | |

| | | | |
|---|---|---|---|
| **Medication Reminder** | | | |
| An option to report medication taking after receiving the reminder or “take” | 95.8% (+) | | |
| An option to delay the reminder so it notifies you later, “snooze” or “postpone” | 91.7% (+) | | |
| An option to report that the medication was not taken after receiving the reminder or “skip” | 95.8% (+) | | |
| **MODULE 3** | | | |
| **Medication Administration Report** | | | |
| A report that shows a percent of doses taken | 70.8% (+) | 62.5% (+) | 50% (+) |
| A report that shows days missed on a daily calendar | 60.9% (+) | 80% (+) | |
| A report that shows days missed on a monthly calendar | 95.8% (+) | | |
| **MODULE 4** | | | |
| **Additional Features** | | | |
| **Drug Information** | | | |
| Record/log of side effects experienced | 70.8% (+) | 88.9% (+) | |
| Drug interactions checker | 75 % (+) | 88.9% (+) | |
| Additional information about the drug (i.e. link to an official drug information source containing information about how the drug works, what side effects are associated with the drug, etc.) | 50% (+) | 61.1% (-) | 50% (+) |
| Overdose warning for maximum daily dose of as needed medications | 83.3% (+) | 83.3% (+) | |
| Record/log of known drug allergies | 91.7% (+) | | |
| Record/log for home monitoring of blood pressure, glucose levels, asthma control, spasticity level, etc. | 62.5% (+) | 55.6% (+) | 60% (-) |
| **Information sharing, storing or privacy** | | | |
| Ability to share medication information from the app with family, friends or caregiver | 91.7% (+) | | |
| Ability to share medication information from the app with provider (physician, pharmacist, nurse, etc.) | 95.8% (+) | | |
| Emergency contact list | 91.7% (+) | | |
| Privacy settings and password protection | 62.5% (+) | 66.7% (+) | 53.3% (+) |
| **Pharmacy information and reminders** | | | |
| Pharmacy locator function to find a pharmacy near you | 79.2% (-) | 72.2% (-) | 71.4% (-) |
| Automatic refill mechanism through your pharmacy | 70.8% (+) | 77.8% (+) | 92.9% (+) |
| Prescription refill reminders | 95.8% (+) | | |
| Doctor appointment reminders | 70.8% (+) | 66.7% (+) | 85.7% (+) |
| **Other** | | | |
| Inclusion of a gaming system with points or rewards for successful utilization | | | 60% (+) |
| Ability to connect with peers through the app to motivate and share adherence | | | 66.7% (-) |
