# Supplementary material for: Identifying Medication Management Smartphone App Features Suitable for Young Adults With Developmental Disabilities: Delphi Consensus Study
Source: JMIR Mhealth Uhealth. 2018 May 23;6(5):e129. doi: 10.2196/mhealth.9527 (PMC5990856; doi:10.2196/mhealth.9527)
Supplement: Multimedia Appendix 4 [file mhealth_v6i5e129_app4.pdf]

Multimedia Appendix 4: Expert comments provided at the end of each module of the questionnaire.

Overall comments

- Provide the patients/caregivers with options to customize their alerts, screens, dashboards, etc. Let them decide which formats are most appealing to them - that may be more complex to implement to it would be more tailored to the patients/caregivers.

- The app should have a feature that the medical professional can access to see if they pushed any buttons/options. Make things easy for patients, a bit hard (and kind of hidden) for medical professionals.

- Help the patient assess the severity of side effects and provide decision support for what the patient can do the next and follow-up for evaluation.

- Decide who the app is designed for. Your phone can't make you be compliant, or smart. A little done well, beats a lot that no one uses except over achieving health care professionals.

- Strive for a simple, clean, consistent interface.

- Simplicity is key. Colors are helpful as well to make it visually appealing and for certain info to catch attention.

Medication List

Display of the list

- Use of graphics such as pictures for times (sunrise suggesting morning) could be useful. Graphics may help those with low literacy. Individuals with disabilities often have low health literacy too.

- Sort for the day - 8am take X, 10am take Y, 4pm take Z and have a "O" that you can touch to indicate it has been taken.

- I think the critical elements are for identification: picture of the pill, name of med used on prescription, how much to take, and when - all very easy to see. Next, would be any instructions for how to take it, and a way to see easily in summary that you've taken the required doses at the required frequency for the day. Perhaps the how much to take could be laid out at each time point, and then you click that time point when you've taken it and that instance is now checked off or something.

- It might be helpful to be able to access all of the different shapes/sizes/colors a medication can look like since they can vary month to month and that can lead to confusion.

- The picture of the pill would be nice IF you could specify the manufacturer as even a generic, same dose can look ENTIRELY different based on the manufacturer. So, it could end up being confusing and lead to errors if not manufacturer specific. Being able to keep track of when you need to renew a script and ease in re-ordering, I think, would be helpful.

- Ability to take a picture of the tablet with a certain backdrop (e.g. on a white piece of paper) for it to be recognized--maybe not this on the med list.

- Print size for name, dose and amount to take when-- must be large enough to see by elderly.

- Include additional 'as needed' medications.

Entering medications

- Too many auto-populate options will end up being more confusing for a user no matter how well intended. So we know that "prednisone" can come in multiple forms, is it valuable to capture this at the risk of user frustration and error?

- Voice recording, maybe? Ideally, there should be a good interoperabilty between the app and the pharmacy system, so the pharmacist can select the list and send it to the individual's app? Why bother the patient? The pharmacist has the list anyway, and less chance to have errors during the information transition process.

- Speech to text if have to manually enter, and a text reader of some sort if unable to read what on the screen.

- I would worry about speech to text because of how badly people mis-pronounce names. Likewise, I worry somewhat that people might mis-type the name and not realize they are listing the wrong med. Though pics can help with that. Having the generic vs brand name is irrelevant. You need whatever name is on the medication that the person is actually taking.

- I think speech-to-text technology is more easily accessed then trying to figure out how to upload a list from the pharmacy. If there is an easy way to upload the list, then I would change that option to very important to ensure that accurate info is entered as easily as possible.

Information and access to information

- Maybe a warning if one medication can counter act another medication.

- Any contraindications (e.g. Don't drink with this med, don't take with grapefruit, etc.).

- Ability to click on each medication to get further details (instructions on taking with food, time of day, side effects and when to call a doctor, other meds possibly interacting with).

- Access to information on discontinued medications. Not on the front page, but accessible information regarding name, dose, who prescribed, when discontinued, and perhaps why discontinued.

- Important to make a distinction between information that would need to be available to a caregiver and information that is needed for the patient to achieve optimal self-management. The caregiver (and high functioning patients) may need a lot of the info that you have previously described, but the prominent App features needed for the patient to self-manage need to be very simple, with visuals and reminder features etc.

- Guardian or parent contact information. Allergies or advert reaction. Also, perhaps a way to list if any reaching is noted when starting a new medication.

Communication with the Pharmacy

- Ordering online.

End-user input during development

- In general, there is likely too much information, for too many people on the app displays. Is the app for the patient? the drug stir? the medical professional that can get good info from a bunch of "facts". Form should follow function. Listen to what the customer wants, they are the user.

Medication Reminder

Reminder delivery mode

- Audio, Vibrate, or Flashing screen - depends on if the meds are optional or mandatory. If you are after compliance - "Skip" will at least allow you to track that they are non-compliant. Maybe increase alarms to motivate them to take it rather than listen to annoying alarm.

- What is the mode of delivery of the reminder? voice, light, vibration? If the patient skips it, or the patient just did not get the reminder, what will happen? is there any follow up? does the caregiver will be reminded too? can the pharmacist adjust the dose remotely?

- A sketch of the pill (color, size, shape) could be helpful in the reminder screen.

- Geotagging reminders could be helpful as well (for example, to take it while at home, or work).

Took option

- Audio (verbal) reminder and audio output when you hit a button (e.g., "took it").

Skip option

- Do not use the word 'skip', as it seems to give permission to do so, and that is not appropriate for most important drug prescriptions. 'not taken' or some other neutral word will increase compliance.

- An option to flag a caregiver/family member that the medication was skipped via text messaging to another phone/device.

Snooze option

- Snooze option is great, with option for how long to snooze.

- A way of setting a delay reminder, in the event the person is away from phone, or doctor's appointment, etc.

- Are there going to be sounds or vibrations associated with the app, so that if a person hits postpone they can be reminded at a later time?

Medication Administration Record

Administration record presentation

- Reporting will depend upon how many meds the patient is taking - 25 pills a day vs 4 pills a day are very different. If very many meds, daily dashboard may be helpful, quite a few - then weekly, not that many, dashboard for month may be enough. Rather than showing days missed, show days taken so the focus is on adherence and getting all the days marked colored. Show how they compared with other patients - or find some motivational framework that will help promote adherence.

- Maybe break adherence dashboard into daily, weekly, monthly, yearly adherence based on how successful the patient is in taking meds. I like the competitive or gaming angle - if it can be leveraged with peers, or maybe some other motivational reward.

- I like graphs that indicate % taken. Lisinopril ||----89%----| ||; Aspirin ||------100%-----||.

- Give them feedback - e.g. you took 15 out of 19 pills this week or you scored 98% medication compliance this week/month/year.

- Frequency of additional 'as needed' medications (pain medications; extra pulmonary, etc. to share with the medical team).

- How many doses per day need to be taken/are taken.

Export adherence report

- The ability to export a graph to email or even within the app would be great - some graphic representation over time would be helpful to be able to take to the doctor and show patterns (is it only missed on weekend, other factors, etc.).

Reasons for missed doses

- Is there a place or space for recording (self-report, a note) why the dose is missing? And possibly what actions were taken after missing the dose?

- Being able to record WHY a med was skipped IE: had nausea, didn't think could keep down.

- Tying a narrative to missed doses as a comment section would be helpful (i.e. didn't take medication because forgot to eat lunch and have to take with meals, etc.).

Strategies to ensure medications are taken

- Scan the pill in your hand or bottle when you take the pill - will give the time, location, pill type, (alert - you already took that pill - you should take pill Y, not pill X at this time). This will close the loop and if it makes it from the hand to mouth, it will be accurate.

- Take a picture of the pill that the patient is ingesting - you can tell when, if it is the right pill, location taken, close the loop more (still they could take a picture and not take it…).
